# Intramedullary Nailing of Concurrent Bilateral Tibial Stress Fractures Followed by Unilateral Tension Plating for a Nonunion in a Vitamin D-Deficient Elite Football Player

**DOI:** 10.7759/cureus.30537

**Published:** 2022-10-21

**Authors:** Dimitris Vasileiou, Argyris C Hadjimichael, Kyriakos Kakavelakis, Andreas Panagiotou, Christos Zafeiris, Efstathios Chronopoulos

**Affiliations:** 1 Orthopaedics, Mediterranean Hospital of Cyprus, Limassol, CYP; 2 Orthopaedics, Imperial College Healthcare NHS Trust, St Mary's Hospital, London, GBR; 3 Sports Traumatology, Mediterranean Hospital of Cyprus, Limassol, CYP; 4 Orthopaedics and Spine Surgery, Metropolitan General Hospital, Athens, GRC; 5 Orthopaedic Surgery, National and Kapodistrian University of Athens, Athens, GRC; 6 Orthopaedic Surgery, Konstantopoulio General Hospital - Nea Ionia, Athens, GRC

**Keywords:** non-union, intramedullary nailing, tibia, bilateral, stress fracture

## Abstract

Stress fractures consist of a type of bone fracture that occurs due to repetitive mechanical stress instead of acute forceful injuries that cause common fractures. They are quite common among athletes at all competition levels and in army recruits who are expected to undergo extremely demanding exercises. While stress fractures can occur in any long bone, they are usually associated with the most common weight-bearing sites of lower extremities such as phalanges, metatarsals, tarsal bones, the tibia, and fibula. In this study, we report the surgical management of a 23-year-old African football player who sustained concurrent bilateral anterior cortex tibial midshaft fractures. His initial symptom was persistent subacute pain in both tibias. The initial conservative treatment was not successful and the patient was surgically treated with bilateral tibial intramedullary nails. However, the right tibia subsequently developed nonunion. Both intramedullary nails were removed and a tension plate was applied with an autologous iliac crest graft on the right tibia. Further blood test analysis revealed a significant vitamin D deficiency. The purpose of this article is to report different outcomes of the same primary surgical treatment for concurrent bilateral tibia stress fracture syndrome in an elite athlete due to vitamin D deficiency. To our knowledge, this is the first study that highlights the necessity of revising one of the intramedullary nailed concurrent tibia stress fractures with a tension plate and autologous graft to treat the established nonunion in an elite football player.

## Introduction

Stress fractures result from repetitive cyclical mechanical loads that are applied either in normal bones, causing fatigue fractures, or in abnormal bones, leading to insufficiency fractures [1]. Fatigue stress fractures mostly occur in young active persons such as athletes and military recruits who engage in highly demanding physical activities [1]. On the contrary, insufficient fractures occur mostly in elderly patients secondary to osteoporosis, sarcopenia, and frailty [1]. The physical history of fatigue stress fractures begins with repetitive elastic deformations that continue as plastic deformities causing tiny microfractures [2]. Subsequently, the insufficient bone healing of these repetitive microfractures as well as the absence of bone remodeling lead to occult non-complete fractures [2]. Consequently, stress fractures appear as chronic subclinical injuries with moderate symptoms in the early stages. However, inadequate stabilization, repetitive movements, and short recovery make them prone to re-fractures [2].

There are numerous risk factors associated with the increased incidence of stress fractures in athletes. Several demographic factors, such as female gender, white ethnicity, increased height, and advanced age, are associated with fatigue stress fractures. In addition, lifestyle habits such as long time intervals between physical inactivity and sudden increase in sports activities have a significant impact on young athletes and may lead to stress fractures. Furthermore, metabolic bone diseases causing accelerated bone turnover and bone loss such as low bone mineral density (BMD), 25(OH) vitamin D value lower than <75.8 nmol/L, hyperthyroidism, and hypoparathyroidism are correlated with an increased incidence of stress fractures in elite athletes [3,4].

Once a lower limb stress fracture is diagnosed, providing appropriate treatment is crucial for ensuring an early return to sports activities, especially in athletes who wish to regain their pre-injury level of athletic performance. Besides, there is evidence to suggest that conservative management of midshaft tibial fractures in athletes is associated with higher complication rates and a decreased probability of returning to sports activities compared to surgical treatment [5]. Multiple surgical options are available for the treatment of tibial stress fractures, such as tension plating, intramedullary nailing (IMN), and even removal/grafting or drilling/revascularization at the fracture site [5]. Based on the literature, conservative treatment requires 12 months for healing and a return to sports activities compared to 11 weeks to four months for tension plating or IMN [5,6]. Certainly, early mobilization and recovery can be achieved by using the IMN technique. However, residual and disabling postoperative anterior pain may develop in 47.4 and 73.2% of these cases at the entry point of the nail [7]. Also, tension plating may further disturb the biology at the fracture site. Subsequently, the extended period of immobilization when using open reduction and internal fixation (ORIF) techniques may cause muscle atrophy and imbalance leading to a prolonged rehabilitation period [7]. In terms of postoperative outcomes and complications, IMN and ORIF with tension plates have no significant difference regarding operation time, radiation time, and nonunion and union time [8]. However, it has been shown that IMN leads to higher functional improvement and reduced superficial infection rates postoperatively [9]. Moreover, it has been found that the mean time for returning to sports activities is six months if either IMN or plates are chosen for the surgical treatment of anterior tibial stress fractures [10].

The primary aim of our treatment plan was to ensure the immediate recovery of an African elite male athlete who had sustained synchronous bilateral midshaft tibia fractures so that he can return to sports activities at a high level. Our patient had an almost identical stress fracture in the shaft of both of his tibias and the initial surgical technique was exactly the same for both. Nevertheless, the choice of treatment should always be adjusted to the needs of the patient and may be totally different for the same medical problem, as we can ascertain from our case. The most significant aspect of our patient's case was that despite the similarity of the fractures and the treatment with the same technique (IMN), the outcomes were different. Unfortunately, the right tibial fracture was not united and had to be revised using a tension plate and autologous bone graft.

## Case presentation

A 23-year-old African football player with no significant past medical history was referred to our hospital two years ago with complaints of persistent dull pain and swelling on the midshaft of his left tibia. He denied smoking and alcohol consumption or taking any kind of medication. He had started playing amateur football at the age of 12 years, and professionally for the last eight years. His position in the field was one of the most demanding ones as he was a forward player, which meant that he had to move constantly and switch places continuously. On the first admission, the X-rays of his lower limb and adjacent joints were unremarkable. Initially, rest and taking time off from football games were suggested and physiotherapy was proposed in order to alleviate the burden on lower limbs from heavy mechanical loads during football games. Initially, a diagnosis of a muscle sprain was considered to be the most appropriate. In addition, he was prescribed non-steroid anti-inflammatory drugs (NSAIDs) and was advised to avoid excessive weight-bearing by using crutches for two weeks. Subsequently, the patient continued his sports activities and attended routine intensive workouts after the improvement of his initial acute symptoms.

**Figure 1:**
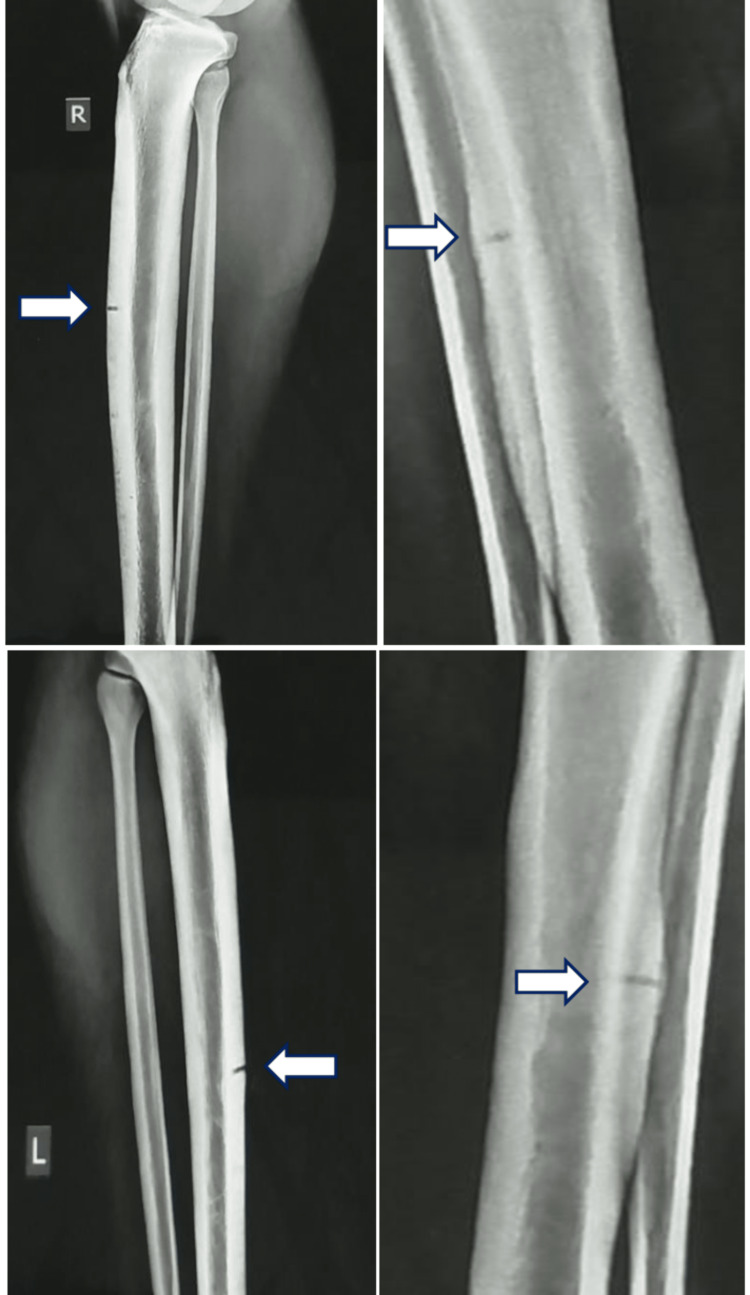
Plain radiographs depicting the synchronous bilateral midshaft stress fractures at the anterolateral cortex of the patient’s tibias

However, one year later, the patient was re-admitted to the hospital with exacerbated intolerable pain bilaterally on his tibias. The X-rays depicted a concurrent transverse cortical break with a typical V-shaped defect in the anterolateral cortex at the midshaft of both tibias, as shown in Figure 1. Meanwhile, the patient’s laboratory blood tests revealed a significant deficiency of 25(OH) vitamin D with levels as low as 6 ng/ml. Therefore, the patient was prescribed a daily supplement of 800 IU 25(OH)D and 2000 mg calcium [4]. Regarding the very low preoperative levels of 25(OH)D, which were 6 ng/ml, very slow progress was observed to achieve normal levels of up to 45 ng/ml in approximately two months after IMN of both tibias (Table 1). The patient’s BMD was further examined by dual-energy X-ray absorptiometry (DEXA) at L1-L4 lumbar spine vertebral bodies, which was found to be 1.453 g/cm^2^ (adult T-score: 1.9 and Z-score: 1.0), as well as at the neck of his non-dominant (right) hip, which was found to be 1.662 g/cm^2^ (adult T-score: 4.6 and Z-score: 3.1). Consequently, the BMD for both L1-L4 and right femoral neck returned to normal levels.

**Table 1:** Laboratory test results IMN: intramedullary nailing; PTH: parathyroid hormone; TSH: thyroid-stimulating hormone; HCT: hematocrit; Hb: hemoglobin

Serum levels	Preoperative	1-month post-IMN	2 months post-IMN
25(OH) vitamin D (normal: >30 ng/ml)	6↓↓	18.0↓↓	45.3
Total testosterone (normal: 3-10 ng/ml)	3.25	5.60	5.50
PTH (normal: 15-65 pg/ml)	38.5	43.2	44.8
TSH (normal: 0.27-4.2 uIU/ml)	1.08	2.2	1.9
HCT (normal: 42-54%)	38.10↓	42	43.4
Hb (normal: 13.5-18.0 g/dl)	12,60↓	14.2	14.4

**Figure 2:**
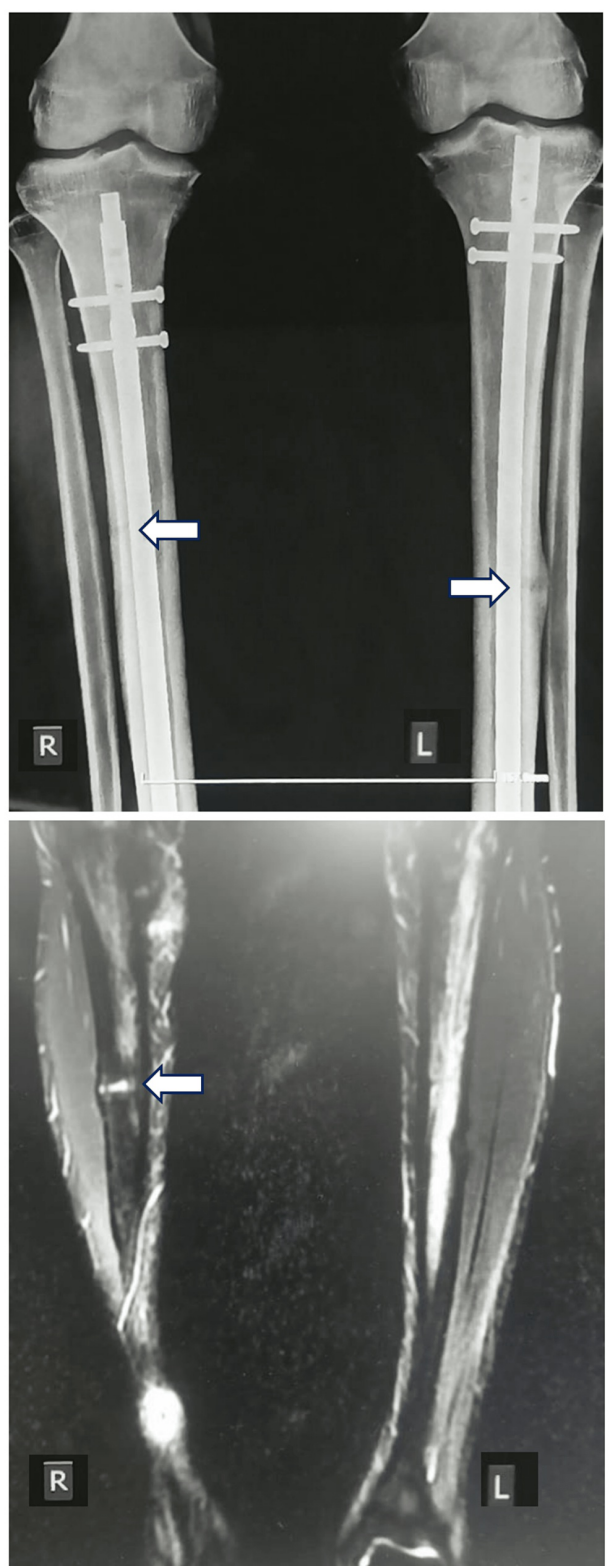
Primary surgical treatment with IMN and evidence of nonunion Top: plain radiographs for both tibias 10 months after the placement of intramedullary nails; right tibia: dreaded black line indicating nonunion; left tibia: bone healing with the formation of hypertrophic mature callus. Bottom: fat-suppressed coronal T1-weighted MRI scan revealing a persistent fracture line (arrow) at the midshaft of the right tibia IMN: intramedullary nailing; MRI: magnetic resonance imaging

Subsequently, the patient was treated with conventional tibial IMN as the primary goal was to ensure early mobilization and quick return to competitive football. Both operations were successful and uneventful. The patient eventually returned to his profession six months postoperatively and continued to play football at the professional level. However, at 10 months postoperatively, the patient started experiencing residual pain in his right tibia. The left tibia was completely painless with radiologic evidence of full secondary bone healing and formation of hypertrophic mature callus. On the contrary, the X-ray of his right tibia showed a dreaded black line indicative of nonunion with segmental bone defect at the site of the previous stress fracture, as shown in Figure 2. The patient was further examined with an MRI of both of his tibias, which confirmed right tibial nonunion and proper left tibial healing (Figure 2).

**Figure 3:**
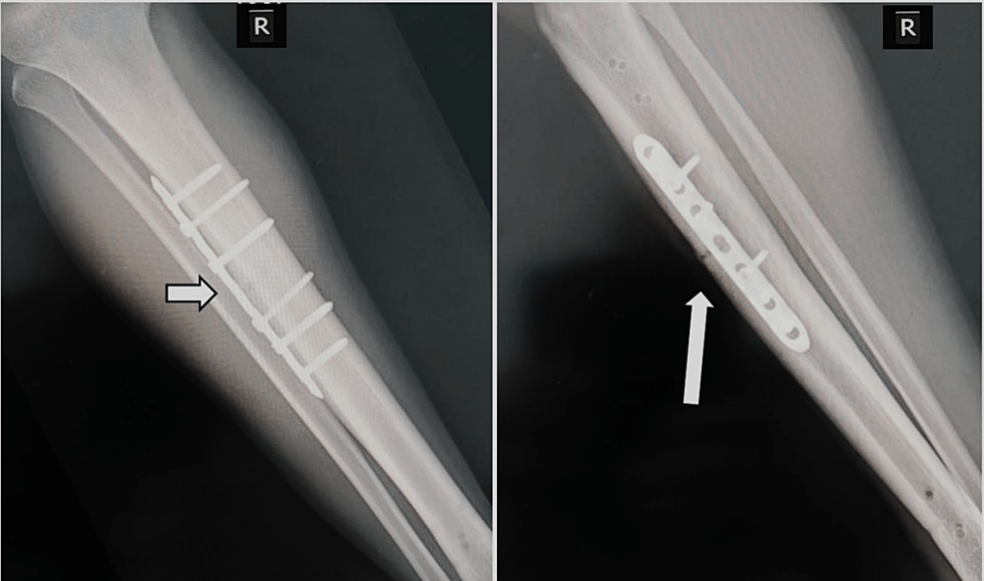
X-ray of the revised nonunited right tibia The images show the tension plate and autologous iliac crest graft on the right tibia with adequate bone healing six months postoperatively

Consequently, both intramedullary nails were removed and a compression technique was performed in the nonunited site. The main aim was to achieve primary bone healing of the nonunited right tibia midshaft stress fracture at that stage. Simultaneously, 25(OH)D levels returned to normal levels, up to 45.3 ng/ml, supporting the bone healing process at that stage. Intraoperatively, the nonunited site was debrided, autologous grafting from the pelvic region was added, and a 7-hole tension plate with 4-locking and 2-non-locking screws was applied for fracture stabilization. The postoperative plan included mobilization and full weight-bearing at six weeks with intensive physiotherapy to achieve muscle strength and early recovery. Eventually, the patient adequately recovered and returned to sports activities six months after his last surgery. He was free of symptoms with X-rays revealing sufficient bone healing (Figure 3). The patient provided full written informed consent for the publication of this report and the imaging details.

## Discussion

According to most studies, the tibia is the most common anatomic location for stress fractures, followed by the tarsal navicular, metatarsal, fibula, femur, pelvis, and spine [11]. To our knowledge, track-and-field football players exhibit the highest incidence of stress fractures among all athletes. Unfortunately, the rehabilitation of stress fractures is not easily achieved due to a variety of risk factors that are not well-documented, such as inappropriate training methods and false repetitive biomechanical loads during games. Furthermore, specific anatomic features such as muscle and bone strength as well as diet and joint flexibility among athletes are likely to contribute to the risk of developing stress fractures [11]. Therefore, early detection and adequate treatment remain crucial to reduce the disability and helping patients return as soon as possible to their regular sports activities. In addition, while anterolateral midshaft tibial fractures are relatively uncommon (4.6%), they are associated with the highest propensity for delayed union, nonunion, pseudarthrosis, and increased risk of transitioning to complete tibial fractures [11].

According to Patel et al., many risk factors increase the propensity for stress fractures to occur [11]. The patient presented in this article had the following ones: excessive physical activity due to his involvement in a professional, demanding track sport such as football; low levels of 25-hydroxyvitamin-D; more than 25 miles of running per week; and sudden increase in physical activities as the fractures were diagnosed after a holiday and absence from sports activities [11]. We believe that athletes should be routinely assessed for 25(OH)D-dependent calcium homeostasis regarding laboratory tests such as parathyroid hormone, 25-OH-D3, calcium, TSH, and creatinine [12]. A deficiency of 25(OH)D has to be corrected with vitamin D and calcium supplements to achieve 25(OH)D blood levels above 30 ng/mL [12].

In the majority of tibial stress fracture cases, the symptoms progress for a mean period of 12 months before the definitive diagnosis is established [13]. In addition, the radiological and clinical union of stress fractures are present at a mean of three and 2.7 months respectively, and a return to sports activities can be achieved four months postoperatively [13]. Despite a low incidence of complications such as nonunion, in the present case, the young athlete developed radiological and clinical findings of nonunion 10 months postoperatively at his right tibia despite the same primary surgical approach.

A four-case study by Borens et al. reported adequate bone healing and rapid return (mean of 10 weeks) to pre-injury competition performance levels in female athletes who had sustained unilateral anterior cortex tibial stress fractures. These patients were treated with tension band plating [14]. Furthermore, for a nonunited stress fracture that was initially treated with IMN in an elite football athlete, Tsakotos et al. preferred the additional placement of a tension band plating without removing the existing IMN nail with great results [15]. According to their report, the use of IMN did not achieve sufficient consolidation at the fracture site even 18 months postoperatively but the complete radiologic union was evident six months after the placement of a tension band plate [15].

The outcomes of surgical treatment for anterior tibial stress fractures were extensively studied by Chaudhry et al. in 2019 [16]. This entailed an exhaustive systematic review involving 12 studies published between 1984 and 2015. According to the authors' findings, the most preferred surgical technique for the treatment of tibial stress fractures was compression plating followed by drilling [16]. However, in the present case, the initial treatment with IMN was considered the most appropriate in line with the findings of Chung et al., who reported excellent outcomes after a combination of vitamin D supplements and static IMN in a young female athlete with concurrent bilateral tibial stress fractures [17]. We hold the view that IMN of synchronous tibial stress fractures remains a good surgical option in young patients as this could help with an earlier return to sports compared to the application of bilateral compression plating at the fracture sites. Despite our efforts to ensure an early recovery, the patient developed a nonunion of the right tibial stress fracture. However, based on the recent literature, this complication occurs in approximately 14.8% of patients who are surgically treated for this type of fracture [16]. In addition, our case report reveals a weakness in the initial management of this patient as laboratory tests were not performed and vitamin D deficiency was not promptly diagnosed during the conservative treatment. We strongly believe that early diagnosis of vitamin D deficiency and administration of the appropriate supplements from the outset itself could have prevented the development of a nonunion after the primary treatment with IMN.

To the best of our knowledge and based on recently published literature, our case report is the first of its kind as it presents different outcomes of bilateral anterior mid-shaft tibia stress fractures that were treated with the same initial method using IMN. Removal of IMN was preferred as we strongly believe that bilateral metalwork and additional placement of tension band plate could impact the return to high-level athletic performance. Eventually, a compression plate was used accompanied by an iliac crest autograft to strengthen the anterior tibial cortex and reduce the risk of recurrent stress fractures.

## Conclusions

Stress fractures must always be included in the differential diagnosis for all young athletes and army recruits who present with chronic pain and swelling at any part of the lower limb. Tibial stress fractures may be treated operatively and nonoperatively. Anterolateral midshaft fractures are the most unusual types compared to the most common variant occurring at the lower third and posterior-medial aspect of the tibias. Some patients diagnosed with tibial stress fractures may suffer from metabolic bone disease or vitamin D deficiency, which requires additional medical interventions to prevent the chronicity of the disease and the complications related to surgical treatment, such as nonunions.

The present case report is the first of its kind in the literature and presents concurrent tibial stress fractures in an elite football player, treated with the same technique (IMN) but with different outcomes, requiring the surgical revision with plating plus autologous grafts to treat a common complication such as a nonunion. Laboratory tests to identify underlying metabolic diseases are strongly recommended preoperatively in similar cases.
